# Improved library preparation protocols for amplicon sequencing-based noninvasive fetal genotyping for *RHD*-positive D antigen-negative alleles

**DOI:** 10.1186/s13104-021-05793-4

**Published:** 2021-09-26

**Authors:** Asuka Hori, Hiroko Ogata-Kawata, Aiko Sasaki, Ken Takahashi, Kosuke Taniguchi, Ohsuke Migita, Akihiro Kawashima, Aikou Okamoto, Akihiko Sekizawa, Haruhiko Sago, Fumio Takada, Kazuhiko Nakabayashi, Kenichiro Hata

**Affiliations:** 1 Department of Maternal-Fetal Biology, National Research Institute for Child Health and Development, 2-10-1 Okura, Setagaya, Tokyo, 157-8535 Japan; 2 Department of Medical Genetics and Genomics, Kitasato University Graduate School of Medical Sciences, Kanagawa, Japan; 3 Center for Maternal-Fetal, Neonatal, and Reproductive Medicine, National Center for Child Health and Development, Tokyo, Japan; 4 Department of Obstetrics and Gynecology, The Jikei University School of Medicine, Tokyo, Japan; 5 Faculty of Medicine, University of Tsukuba, Ibaraki, Japan; 6 Department of Obstetrics and Gynecology, Showa University School of Medicine, Tokyo, Japan; 7 Laboratory of Developmental Genomics, National Research Institute for Child Health and Development, 2-10-1 Okura, Setagaya, Tokyo, 157-8535 Japan

**Keywords:** *RHD*, Cell-free DNA (cfDNA), Non-invasive prenatal testing (NIPT), Amplicon sequencing, Unique molecular identifier (UMI)

## Abstract

**Objective:**

We aimed to simplify our fetal *RHD* genotyping protocol by changing the method to attach Illumina’s sequencing adaptors to PCR products from the ligation-based method to a PCR-based method, and to improve its reliability and robustness by introducing unique molecular indexes, which allow us to count the numbers of DNA fragments used as PCR templates and to minimize the effects of PCR and sequencing errors.

**Results:**

Both of the newly established protocols reduced time and cost compared with our conventional protocol. Removal of PCR duplicates using UMIs reduced the frequencies of erroneously mapped sequences reads likely generated by PCR and sequencing errors. The modified protocols will help us facilitate implementing fetal *RHD* genotyping for East Asian populations into clinical practice.

**Supplementary Information:**

The online version contains supplementary material available at 10.1186/s13104-021-05793-4.

## Introduction

Alloantibodies against Rh antigens represent the main cause of hemolytic disease of the fetus and newborn (HDFN). The D antigen is the most highly immunogenic among Rh antigens. RhD-negative women become sensitized to the D antigen and subsequently produce anti-D antibodies when they carry an RhD-positive fetus. Although anti-D prophylaxis by postnatal and antenatal anti-D Ig administration has been highly successful in reducing the incidence of HDFN worldwide [1], it is unnecessary for RhD-negative women who carry an RhD-negative fetus. Fetal *RHD* genotyping makes it possible to prevent unnecessary anti-D administration in such pregnancy cases. The fetal *RHD* genotyping method widely implemented in western countries is designed to detect the presence or absence of the *RHD* wild-type allele of fetal origin in the plasma of RhD-negative pregnant women, over 99.9% of whom are homozygous for *RHD* deletion alleles in Caucasian populations. Because of relatively high frequencies of *RHD*-positive RhD-negative alleles, RHD*01EL.01 and RHD*01N.04, among RhD-negative individuals (9.0% and 2.9%, respectively, in the Japanese population), the same genotyping method was not applicable in East Asian countries. The RHD*01EL.01 allele contains a single nucleotide variant at the last nucleotide of exon 9 (c.1227G/A), which likely disrupts normal splicing [2]. The RHD*01N.04 allele is a hybrid allele, in which exons 3–9 of the *RHD* gene are replaced with those of *RHCE* [3].

**Fig. 1:**
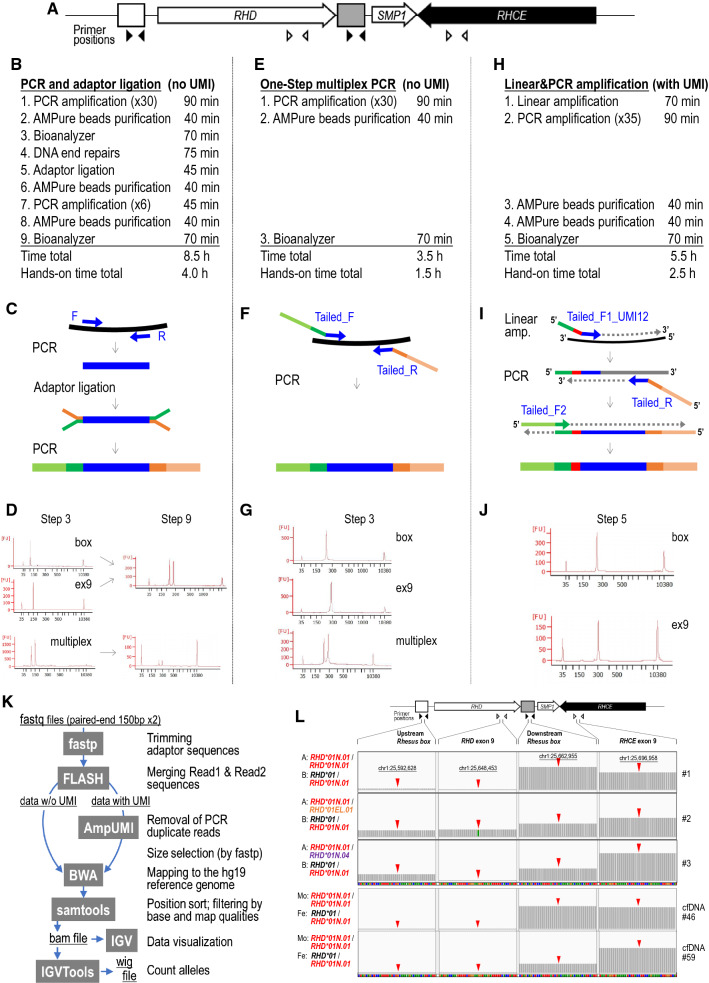
Conventional and newly developed library preparation protocols for amplicon sequencing-based noninvasive fetal *RHD* genotyping. **A** Genomic organization of the *RHD/RHCE* locus. Open and closed boxes indicate upstream and downstream *Rhesus boxes*. Closed and open arrowheads indicate PCR primer pairs to amplify 105-bp intervals in *Rhesus boxes* and 148-bp intervals spanning the exon 9 and the intron 9 of *RHD* and *RHCE* genes, respectively. Amplicon-seq libraries without UMI can be prepared by our conventional ligation-based protocol (**B,** **C**) and the one-step multiplex PCR protocol (**E,** **F**) established in this study. Amplicon-seq libraries with UMI can be prepared by the newly established linear and PCR amplification protocol (**H,** **I**). Approximate total and hands-on time (**B,** **E,** **H**), diagrams for experimental procedures (**C,** **F,** **I**), and representative electropherograms of intermediate PCR products and/or final libraries (**D,** **G,** **J**) are shown for three protocols. In the panels **C**, **F**, and **I**, genomic DNA is shown by a black line; PCR primers that target genomic DNA sequences are shown by blue arrows; adaptor sequences are shown in dark/light green/orange; 12-base UMIs are shown in red. Index sequences to de-multiplex sequence reads after sequencing of pooled libraries are omitted for simplicity. Panels **D**, **G**, and **J** show representative electropherograms of *Rhesus box* amplicons (“box”), for *RHD/RHCE* exon 9 amplicons (“ex9”), and for multiplex amplification by two pairs of primers (“multiplex”). Horizontal and vertical axes of each electropherogram represent fluorescent intensity and DNA size (bp), respectively. Lower and upper marker peaks are present at the positions of 35 bp and 10,380 bp, respectively. In our conventional [4] and one-step PCR protocols (**C**, **F**), multiplex PCR using two pairs of primers gave rise to specific amplification of target genomic intervals (**D**, **G**). **K** A data analysis workflow showing the informatics tools used and their step-by-step functions. **L** Bam coverage tracks of the *RHD* genotyping results by the one-step PCR protocol (**F**) for three combinations of the mixtures of genomic DNA (combinations #1, #2 and #3 in Additional file 2: Table S2) and for two cfDNA samples (#46 and #59) from RhD-negative pregnant women (Table 1) visualized using IGV. The mapped read patterns indicated that the *RHD* genotypes of the mother (Mo) and the fetus (Fe) were RHD*01N.01/RHD*01N.01 and RHD*01/RHD*01N.01, respectively, in both cases. The vertical ranges of mapped read numbers were 0–25,000 for the top three panels, 0–40,000 for cfDNA#46, and 0–150,000 for cfDNA#59

We have recently established an amplicon-based noninvasive fetal genotyping method that distinguishes the wild-type *RHD* allele not only from the *RHD-*negative D antigen-negative allele (the *RHD* deletion allele), but also from *RHD*-positive D antigen-negative alleles [4]. This method requires PCR amplification from four genomic intervals, upstream and downstream *Rhesus boxes*, *RHD* exon 9, and *RHCE* exon 9. Because of extremely high sequence similarities between two *Rhesus boxes* and between *RHD* exon 9 and *RHCE* exon 9, we designed two primer pairs to amplify these four regions (Fig. 1A, Additional file 1: Fig. S1). One primer pair perfectly matches with two genomic intervals, upstream and downstream *Rhesus boxes,* and amplifies 105-bp PCR products. The other primer pair also perfectly matches with two genomic intervals, *RHD* exon 9 and *RHCE* exon 9 regions, and amplifies 148-bp PCR products. The 105-bp PCR products contain one base difference that distinguishes two *Rhesus box* sequences. The 148-bp PCR products contain two base differences that distinguish two genes, and also cover the point mutation site in exon 9 of the RHD*01EL.01 allele (c.1227G/A) that distinguishes it from the wild-type allele (RHD*01). Although two regions are co-amplified with one primer pair, attachment of adaptor sequences to the PCR amplicons followed by NGS allowed us to accurately map each of the co-amplified sequences to its origin because of the one or two base differences between two regions, in the data analysis procedure [4].

In this study, we simplified our fetal *RHD* genotyping protocol by changing the adaptor attachment method from ligation (Fig. 1B, C) to a one-step PCR (Fig. 1E, F). We also evaluated whether introduction of unique molecular indexes (UMIs) [5, 6] (Fig. 1H, I) improves the quantitative accuracy in measuring the ratios of *RHD* alleles in cfDNA.

## Main text

### Material and methods

#### Blood collection and DNA extraction

Cell-free DNA in maternal plasma was extracted using the Mag MAX Cell-Free DNA Isolation Kit (Thermo-Fisher Scientific, A29319) as described previously [4]. Individuals with three major RhD-negative genotypes, RHD*01N.01/RHD*01N.01, RHD*01N.01/RHD*01.04, and RHD*01N.01/RHD*01EL.01 in the Japanese population, and those with two RhD-positive genotypes, RHD*01/RHD*01 and RHD*01/RHD*01N.01, were identified as described previously [4].

#### Preparation of amplicon sequencing libraries by one-step PCR (Fig. 1E, F)

A tailed-forward primer (“Tailed_F”) contains the Illumina forward (P5) adaptor sequence (70 bases) including an 8-bp index followed by a target-specific forward primer sequence (20 or 22 bases) at the 3′ end (90 or 92 bases in total). A tailed-reverse primer (“Tailed_R”) contains the Illumina reverse (P7) adaptor sequence (66 bases) including an 8-bp index followed by a target specific reverse primer sequence (25 or 29 bases) at the 3′end (Additional file 2: Table S1). PCR was performed with 2 ng of a mixture of genomic DNA of two individuals (or cfDNA) using 0.5 unit of Q5 Hot Start High-Fidelity DNA Polymerase (M0493, NEB) according to the manufacturer’s instruction in a 25 µL reaction at the following final concentrations: 1 × Q5 reaction buffer, 0.2 mM dNTPs, 0.5 µM each of the tailed primers. The thermal cycling conditions used were 98 °C for 30 s, 30 cycles of 98 °C for 10 s, 64 °C for 30 s, and 72 °C for 30 s, and 72 °C for 2 min. Twenty out of the 25 µL reactions were purified using 0.9 times the volume (18 µL) of Agencourt AMPure XP (A63881, Beckman Coulter) and eluted with 10 µL distilled water. The purified PCR products, i.e., the final libraries, were electrophoresed using the High Sensitivity DNA Kit on a 2100 BioAnalyzer (Agilent) to confirm their sizes, and subjected to paired-end sequencing (151 bp × 2) on a MiSeq system (Illumina) using MiSeq Reagent Kit v2 Nano.

#### Preparation of amplicon sequencing libraries with unique molecular identifier (UMI) sequences (Fig. 1H, I)

Cell-free DNA was concentrated using a centrifugal evaporator (MicroVac MV-100, TOMY) when necessary. Linear amplification was performed in a 23 µL scale reaction consisting of 8 ng of a mixture of genomic DNA of two individuals in 10.35 µL, 1.15 µL of 0.4 µM “Tailed_F1_UMI12” primer (final concentration of 20 pM, Additional file 2: Table S1), and 11.5 µL of Q5 Hot Start HiFi PCR Master Mix (M0543, NEB). The thermal conditions for linear amplification were 98 °C for 2 min, 57 °C for 15 min, 61 °C for 15 min, and 65 °C for 5 min. Subsequently, 1.0 µL each of 10 µM “Tailed_F2” and “Tailed_R” primers, and 25.0 µL of Q5 Hot Start HiFi PCR Master Mix were added to the 23 µL reaction and mixed by pipetting. The resultant 50 µL reactions were subjected to PCR amplification with the following conditions for *Rhesus boxes*: 98 °C for 1 min; 35 cycles of 98 °C for 10 s, 64 °C for 30 s, and 68 °C for 45 s; 68 °C for 5 min, and for *RHD/RHCE* exon 9: 98 °C for 1 min; 35 cycles of 98 °C for 10 s, 66 °C for 30 s, and 70 °C for 45 s; 70 °C for 5 min. Twenty out of the 50 µL reactions were purified using 0.9 times the volume (18 µL) of Agencourt AMPure XP repeated twice, and eluted with 10 µL distilled water. The final libraries were electrophoresed and sequenced as described above.

#### Data analysis (Fig. 1K)

For amplicon libraries without UMIs, fastq files were generated using bcl2fastq V2.20.0.422 (Illumina), and trimmed for adaptor sequences using fastp ver.0.21.0 [7]. Read 1 and read 2 sequences were merged using FLASH ver.1.2.11 [8] with a parameter of “-max-mismatch-density = 0”, and the merged sequences were filtered by their expected size (105 bases for *Rhesus boxes* and 148 bases for *RHD/RHCE* exon 9) to remove reads with unexpected sizes (such as primer dimers and PCR artefacts). The merged sequences were mapped to the hg19 reference genome using “bwa aln” and “bwa samse” commands of bwa-0.7.17 [9]. By using samtools -1.4.1 [10], the resultant bam file was sorted in a positional order and filtered by base quality scores (cutoff 25) to remove low quality reads, by mapping scores (cutoff 23 for *Rhesus boxes* and 37 for *RHD/RHCE* exon 9) to select uniquely mapped reads. The mapped read numbers were counted for each of the four bases at each nucleotide position of the amplicons using IGVTools_2.3.94 (https://software.broadinstitute.org/software/igv/igvtools) (igvtools count-w 1-bases), and output as a.wig file. Subsequently, the numbers of G at chr1:25,592,628 and of A at chr1:25,662,955 were extracted as read counts of the upstream and the downstream *Rhesus boxes*, respectively. The numbers of G and A at chr1:25,648,453 as the read counts of the wild-type allele and the c1227G > A allele of *RHD* exon 9, and the number of C at chr1:25,696,958 as the read count of *RHCE* exon 9 were extracted. The mapping results were further examined for the existence of unexpected variants or potential sequence errors by visualizing the bam file data for four regions corresponding to the amplicons using IGV (https://software.broadinstitute.org/software/igv/), and by inspecting the minor allele frequency of each nucleotide position of the amplicons using the text data (.wig file) generated by IGVTools. For amplicon libraries with UMIs, fastq generation, adaptor trimming, and merge of paired reads were performed as described above. The resultant merged sequences were further processed with AmpUMI.py 1.2 [6] to remove PCR duplicate reads using 12-base UMI sequences located at the beginning of the original read 1. The sequence reads after removing PCR duplicates were further processed as described above.

The.wig file data were used to calculate the ratio of the read number containing the bases other than the reference base to the total read number at a single nucleotide (i.e., error ratio). These read numbers were initially counted for each nucleotide of the four amplicon regions (upstream and downstream Rhesus boxes, *RHD* exon 9, and *RHCE* exon 9). Error ratios were subsequently calculated using the total numbers for the positionally identical bases between the upstream and downstream at *Rhesus box* amplicons (for 105 positions) and between *RHD* exon9 and *RHCE* exon 9 amplicons (for 147 positions excluding the position of the c.1227A > G variation at chr1: 25,648,453).

### Results and discussion

We established one-step PCR conditions (Fig. 1E–G) and tested on twelve combinations of genomic DNA mixtures of two individuals (A and B) at a 10:1 ratio, which served as approximation models of cfDNA from RhD-negative pregnant women. “A” corresponds to the mother and is any of three RhD-negative genotypes (RHD*01N.01/RHD*01N.01, RHD*01N.01/RHD*01EL.01, or RHD*01N.01/RHD*01N.04), and “B” corresponds to the fetus and is any of four genotypes (one RhD-positive genotype [RHD*01/RHD*01N.01] or three RhD-negative genotypes). These twelve combinations cover 93.6% of possible genotype combinations of a fetus and an RhD-negative pregnant woman in the Japanese population. The observed ratios of amplicons from *Rhesus boxes* and from *RHD/RHCE* exon 9 were mostly consistent with the expected ratios (Additional file 2: Table S2). The reliability for estimating the fetal RhD type of the newly established one-step PCR protocol was confirmed to be comparable with that of the conventional protocol (Fig. 1B–D) [4]. Examples of mapped read data visualized by IGV are provided (Fig. 1L).

UMIs have been used to confidently detect PCR duplicates in NGS applications [6], and have been shown to reduce sequencing error rates and to increase analytical specificity in various studies, including those for NIPT [11, 12]. We established linear and subsequent exponential amplification conditions to introduce 12-base UMIs to the amplicon libraries (Fig. 1H–J), and tested the protocol on the twelve combinations of genomic DNA mixtures described above (Additional file 2: Table S3). The results we obtained by the UMI protocol with our standard filtering conditions were almost completely free of erroneous reads, and were not suitable to assess the efficacy of UMI to reduce errors. When we analyzed the same dataset with less stringent filtering conditions, we confirmed that the UMI-based removal of PCR duplicates lowered the ratio of erroneous reads for the majority of cases (data not shown). We observed a stronger tendency of the amplification bias towards *RHCE* exon 9 over *RHD* exon 9 in the dataset by the linear & PCR amplification protocol (Additional file 2: Table S3) than that by one-step PCR (Additional file 2: Table S2). Further optimization of the linear and the PCR amplification conditions is required to minimize the observed amplification biases.

PCR and sequencing errors are inherent in the current NGS technologies [13]. We calculated the ratios of such errors presumably generated during library preparation and sequencing procedures and retained after applying read filtering conditions in the data analysis procedure. When the mapped read data of twelve samples in Additional file 2: Table S2 were analyzed, the highest error ratios were 0.06% and 0.11% for *Rhesus box* amplicons and *RHD/RHCE* exon 9 amplicons, respectively (Additional file 1: Fig. S2). Although potential PCR or sequencing errors at the nucleotide positions to distinguish the origin of each sequence read were observed at low frequencies, as shown in red in Table 1, Additional file 2: Tables S2 and S3, their ratios were below the calculated background levels except for one case (0.19%) detected for the fourth combination of genomic DNA mixture (Additional file 2: Table S2). Such a high error ratio indicates the possibility of carry-over contamination from previous PCR assays. UMI is expected to be useful as a means to remove such contaminated reads.

**Table 1:** Mapped read ratios of one-step PCR amplicons from cell-free DNA of RhD-negative pregnant women

Cell-free DNA from a RhD-negative pregnant woman		Amplicons from *Rhesus boxes* (Primers: RHbox_Tailed_F/R)	Amplicons from *RHD/RHCE* exon 9 (Primers: RHD/RHCE_exon9_Tailed_F/R)
		Observed ratio and read counts (upstream/downstream)	Observed ratio and read counts (*RHD*_wt/*RHD*_var/*RHCE*)
Cell-free DNA #46 (8 weeks)		1.88%/98.12% (416/21,694)	1.33%/0%/98.67% (281/0/20,914)
Cell-free DNA #59 (28 weeks)		3.60%/96.40% (813/21,754)	2.05%/0.002%/97.95% (1,253/1/59,758)

The gestational week of blood sampling is shown in parentheses

In NIPT methods, fetal fraction (FF), the ratio of fetal DNA in cfDNA in maternal plasma, has been recognized as the most critical factor for their diagnostic accuracy [14]. The sensitivity of trisomy 21 detection dropped from 99 to 75% when FF was below 8% [15]. In our method, when the RHD*01 allele is detected in cfDNA from maternal plasma, it determinately demonstrates that the fetus is RhD-positive. On the other hand, when the RHD*01 allele is undetected, it leaves two possibilities, namely, that the fetus is RhD-negative and homozygous for the *RHD* deletion (RHD*01N.01) allele, or that the assay failed to detect an RhD-positive allele of the fetus due to a low FF. Accurate determination of the target molecule number in a genotyping PCR reaction through UMI-based removal of PCR duplicates helps us better presume which one of the possibilities is more likely.

### Limitations

The limitation of our amplicon-sequencing-based fetal *RHD* genotyping is that the method by itself cannot determine FF, which is common to all amplicon-based NIPT methods. To complement this limitation, we plan to adopt an amplicon-sequencing method for multiple SNPs [16] when FF of a cfDNA sample needs to be precisely determined.

## Supplementary Information

**Additionalfile1:FigureS1.** Genomic organization of the RhD-positive allele (RHD*01) and three major RhD-negative alleles (RHD*01N.01, RHD*01EL.01, and RHD*01N.04). The genomic positions of PCR primers targeted for Rhesus boxes (closed arrowheads) and for the exon 9 regions of the *RHD* and *RHCE* genes (open arrowheads) are shown for each allele. The nucleotide bases that distinguish the amplicons from the upstream and the downstream Rhesus boxes (G at chr1:25,592,628 and A at chr1:25,662,955) are shown. The nucleotide bases that distinguish the amplicons from *RHD* exon 9 and *RHCE* exon 9 region (A at chr1:25,648,419 and A at chr1:25,648,515 in the *RHD* exon 9 region, and T at chr1:25,696,992 and G at chr1:25,696,896 in the *RHCE* exon 9) are also shown. The red vertical bar shown in the RHD*01EL01 allele represents the c.1227A>G variation at chr1:25,648,453. **Figure** **S2.** Error ratio plots for *Rhesus boxes* (A) and *RHD/RHCE* exon 9 (B). Error ratios, ratios of the read number containing the bases other than the reference base to the total read number, were calculated using the total numbers for the positionally identical bases between the upstream and downstream at *Rhesus box *amplicons (for 105 positions) and between *RHD* exon9 and *RHCE* exon 9 amplicons (for 147 positions excluding the position of the c.1227A>G variation at chr1: 25,648,453). The results for twelve each amplicon libraries for *Rhesus boxes* (A) and *RHD/RHCE* exon 9 (B) prepared by the one-step PCR protocol (without UMI) (Table S2) were shown. For each nucleotide position, the maximum ratio, the median ratio, and the minimum ratio among twelve libraries are shown in dots (in red, black, and blue, respectively). The gray-shaded regions (nt 1 to 20 and nt 81 to 105 for *Rhesus boxes *and nt 1 to 22 and nt 120 to 148 for *RHD/RHCE* exon 9) correspond to PCR primer sequences. Because of the higher error rates consistently observed in the primer regions than in the internal region, the primer regions were excluded for further analyses. The highest error ratio detected in each type of amplicons is indicated by arrow: 0.060% at nt 37 for Rhesus boxes amplicons and 0.116% at nt 40 for *RHD/RHCD *exon 9 amplicons. The median error ratios for the *Rhesus box *amplicons (nt 21 to 80) ranged from 0.00% to 0.032% and those for the *RHD/RHCE* exon 9 amplicons (nt 23 to 119) ranged from 0.00% to 0.030%.

**Additionalfile2:TableS1.** List of primers. **Table** **S2.** Expected and observed ratios of one-step PCR amplicons from the 12 combinations of the 10:1 mixture of genomic DNAs (A and B). **Table** **S3**. Expected and observed ratios of UMI-attached amplicons from the 12 combinations of the 10:1 mixture of genomic DNAs (A and B).

## Data Availability

The sequence read files (fastq.gz) for amplicon libraries (Additional file 2: Tables S2 and S3) and the shell code for the data analysis procedures (Fig. 1K) are available at DRYAD [17].

## Abbreviations

cfDNA: Cell-free DNA
HDFN: Hemolytic disease of the fetus and newborn
PCR: Polymerase chain reaction
NGS: Next generation sequencing
UMI: Unique molecular identifier
NIPT: Non-invasive prenatal testing
FF: Fetal fraction
