# Supplementary figures and images for: Improved library preparation protocols for amplicon sequencing-based noninvasive fetal genotyping for RHD-positive D antigen-negative alleles
Source: BMC Res Notes. 2021 Sep 26;14:380. doi: 10.1186/s13104-021-05793-4 (PMC8474863; doi:10.1186/s13104-021-05793-4)

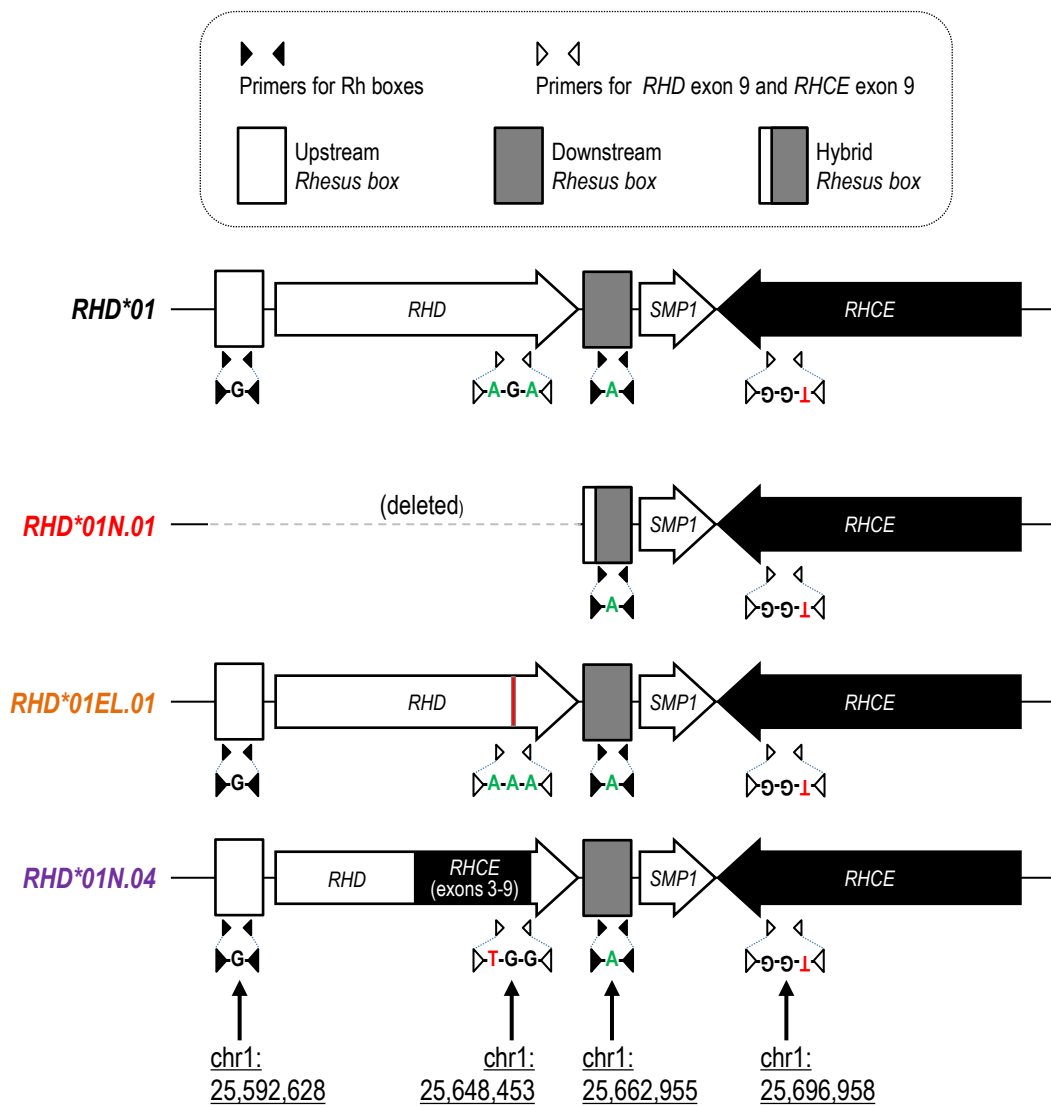

Figure S1 (Hori *et. al.* )

**A*****Rhesus boxes (noUMI, Table S2)***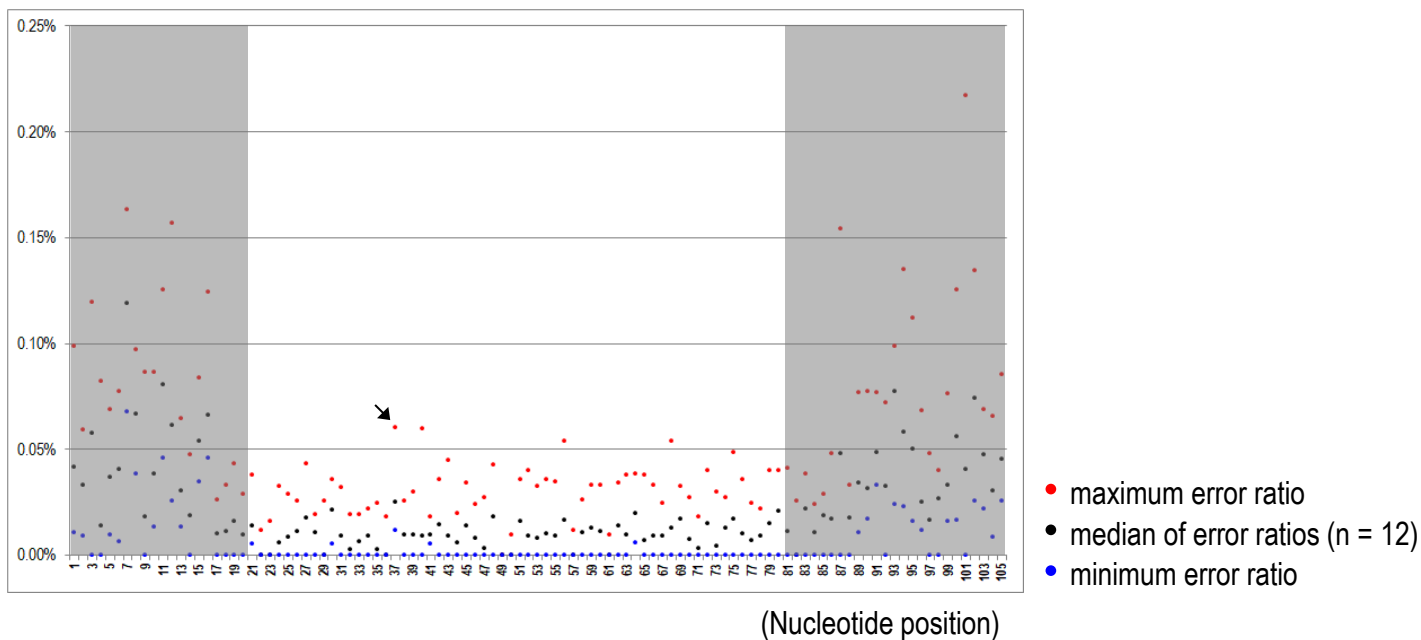**B*****RHD/RHCE exon 9 (noUMI, Table S2)***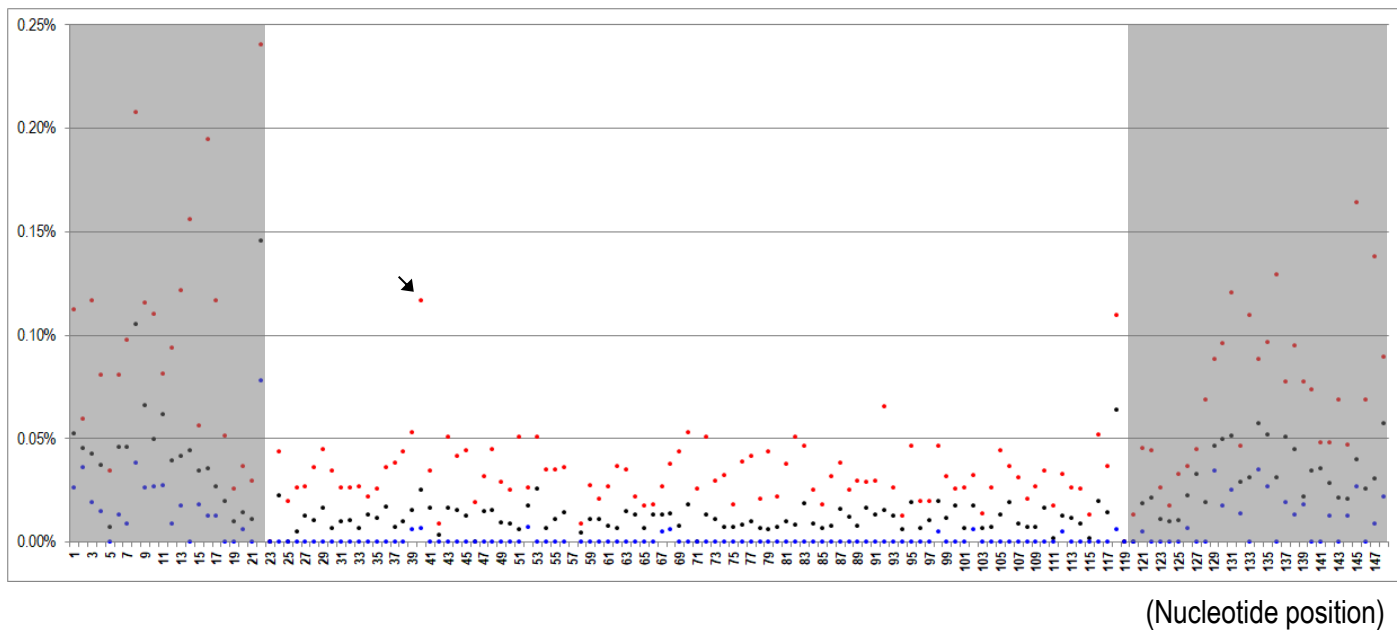**Figure S2 (Hori *et. al.* )**

Supplement: Supplementary file 1 — Additionalfile1:FigureS1. Genomic organization of the RhD-positive allele (RHD*01) and three major RhD-negative alleles (RHD*01N.01, RHD*01EL.01, and RHD*01N.04). The genomic positions of PCR primers targeted for Rhesus boxes (closed arrowheads) and for the exon 9 regions of the RHD and RHCE genes (open arrowheads) are shown for each allele. The nucleotide bases that distinguish the amplicons from the upstream and the downstream Rhesus boxes (G at chr1:25,592,628 and A at chr1:25,662,955) are shown. The nucleotide bases that distinguish the amplicons from RHD exon 9 and RHCE exon 9 region (A at chr1:25,648,419 and A at chr1:25,648,515 in the RHD exon 9 region, and T at chr1:25,696,992 and G at chr1:25,696,896 in the RHCE exon 9) are also shown. The red vertical bar shown in the RHD*01EL01 allele represents the c.1227A>G variation at chr1:25,648,453. Figure S2. Error ratio plots for Rhesus boxes (A) and RHD/RHCE exon 9 (B). Error ratios, ratios of the read number containing the bases other than the reference base to the total read number, were calculated using the total numbers for the positionally identical bases between the upstream and downstream at Rhesus box amplicons (for 105 positions) and between RHD exon9 and RHCE exon 9 amplicons (for 147 positions excluding the position of the c.1227A>G variation at chr1: 25,648,453). The results for twelve each amplicon libraries for Rhesus boxes (A) and RHD/RHCE exon 9 (B) prepared by the one-step PCR protocol (without UMI) (Table S2) were shown. For each nucleotide position, the maximum ratio, the median ratio, and the minimum ratio among twelve libraries are shown in dots (in red, black, and blue, respectively). The gray-shaded regions (nt 1 to 20 and nt 81 to 105 for Rhesus boxes and nt 1 to 22 and nt 120 to 148 for RHD/RHCE exon 9) correspond to PCR primer sequences. Because of the higher error rates consistently observed in the primer regions than in the internal region, the primer regions were excluded for further [file 13104_2021_5793_MOESM1_ESM.pdf]
